# Abstract of Discussion of the Treatment of Deciduous Teeth

**Published:** 1883-03

**Authors:** 


ABSTRACT OF DISCUSSION OF THE TREATMENT OF DECIDUOUS TEETH.

Dr. Field—The one principle that particularly underlies the salvation of deciduous teeth, and at the same time the one most embarrassing circumstance to be met, is the treatment of the pulps of those teeth. The process which nature has provided for their removal is by the absorption of the roots, and the following of the permanent teeth through underneath them. The question therefore arises, are the roots of the deciduous teeth absorbed in cases were the pulps are dead? It has been asserted that they are, and also that they are not. I think Dr. Brophy has studied the pathology of this class of cases, and I would like to hear his views upon that subject.

Dr. Brophy—In my opinion teeth which have lost their vitality are incapable of being absorbed. They may become disintegrated the same as any of the osseous tissues. The cementum may lose its form by a process which we call caries or necrosis, and the dissolved calcium salts may find exit, or possibly be taken up. We know that bones are frequently lost through necrosis followed by liquefaction and gradual absorption. It has been said that it is impossible for nature to remove a dead part by absorption. That, I think, is not true, because we know our bodies are being constantly repaired and renewed and replaced, the old tissues being absorbed. I think then that the roots of deciduous teeth, when devoid of vitality, are liquefied, as in cases of necrosis, gradually broken down and taken away by the absorbents.

There are other matters pertaining to the treatment of deciduous teeth that I might speak of. Sometimes decay commences as early, in the incisors, as the first or second year. I think the better way to treat these cases is simply to sepaıate the teeth by means of a file, cutting them so they may be free, in the manner recommended by Dr. Arthur. This insures a thorough cleansing. With the teeth of my own children I have resorted to that method. One little girl, now seven years old, had caries commence between the central incisors when she was about a year and a half old. I made free separations at that time, and the teeth are now no more diseased than they then were. There is some caries, but the teeth still remain. The great point is to get them so that they will cleanse themselves freely. Now, as to filling cavities in the teeth, the pulps of which are alive, I have good success in using gutta percha. I have also used amalgam, but I have objections to that. Tin or phosphate of zinc are excellent materials, where the cavity can be thoroughly cleansed and dried. In cases where deciduous molars are somewhat decayed, I would pursue the same treatment, making free cuts, so that the apex of the V shaped cut will be at the bottom, and the broad base at the top, in lower teeth, and vice versa in the upper. I speak only of the temporary teeth in those cases. It is very important that a systematic brushing of the teeth should be begun very young.

Dr. Harlan—We are all aware that the absorption of the roots of deciduous teeth is a physiological process; that there is an absorbent papilla in connection with the root and over the tooth that is absorbed. If the papilla of the tooth has been destroyed it is not possible that true absorption follows. In other words, it would seem that the absorption of a pulpless deciduous tooth is not possibie. Another error that has formed a part of some of this discussion is that the incoming tooth produces absorption. I think there is no gentleman present who has not seen an incisor, particularly of the permanent set, come on the inside or outside of a deciduous tooth. This would show that no absorption has taken place. That one fact pretty clearly demonstrates that it is not pressure which produces absorption, because in instances of that kind where the permanent tooth forces its way through on one side of the temporary tooth, there is a great amount of pressure. The late Dr. Dean, of Chicago, has investigated the process perhaps as thoroughly as anyone, and his conclusions are something like what I have stated. There can be no absorption; that there is liquefaction I believe. Dr. Spaulding, of St. Louis, claims that he has filled the roots of deciduous teeth with gold, and afterwards found the little spiculæ of gold projecting into the soft surrounding surface without irritation, when he extracted the teeth. He is the only one who ever claimed that. No one has ever contributed anything to the literature of the treatment of deciduous teeth analagous to it. I shall not dispute it, but I should like to see it. Now, if the pulp of a deciduous molar tooth be destroyed at six years of age and the root filled, the tooth being extracted a year or two later, there would undoubtedly be an excavation in some one of the roots, which would show that there had been an absorption; but that absorption would have taken place previous to the destruction of the pulp. As in fractures of the maxillæ and other accidents, where teeth have been knocked out at those stages, the results of absorption can be seen. It is thus demonstrated that the process of absorption begins very early, as early as the sixth year in the first temporary molars.

Dr. Taft—The question has been raised, "Do the roots of pulpless temporary teeth ever become dissolved away?" Yes; they do become dissolved away, even though the pulp may have been destroyed, whenever the absorbent papille below has not become deteriorated by the debris of the decomposing pulp. If this little tissue, which is in the main independent of the pulp of the temporary tooth, retains its integrity, in a healthy condition, it will go on dissolving away the ends of the temporary teeth, no matter whether the pulp is there or not. But the result would be, in a great majority of cases, that the debris resulting from the death of the pulp would be sufficient to produce disease in, or wholly destroy this little body, and so prevent its further working for dissolution of the roots. I think it is uniformally true that in all cases where inflammation takes place, the function of this body is either very seriously impaired or entirely destroyed.

But are the roots of temporary teeth sometimes dissolved away, even under such circumstances as this? Yes; and sometimes the roots of permanent teeth are dissolved away. They are sometimes honey-combed; they are sometimes dissolved more or less at the end. Occasionally I have seen one-half of the root of a permanent tooth dissolved away, though not by the same process, by which a temporary tooth is dissolved. By a diseased condition, in which the elaboration of a solvent takes place, the root is dissolved away. The same thing takes place with the temporary teeth, but it is a natural physiological action, and not the process that takes place upon the roots of permanent teeth where a sinus forms around them.

As to the treatment of temporary teeth I wish to say a few words. The permanent teeth are influenced somewhat by the forces and processes which act at about the period when the roots of the tempoary teeth are taken away. A temporary tooth may have a serious effect sometimes upon the permanent tooth which succeeds it. Sometimes the crown of a permanent tooth will appear with the enamel roughened all over its surface, and sometimes dissolved. Sometimes you can scrape off the enamel, it is so nearly dissolved. These conditions are always due to the failure in the developing process. This absorbent papilla, this carneous body, is diseased, and its utility perverted or destroyed altogether. The crown of the permanent tooth, therefore, comes in contact with the root of the temporary tooth, and so this roughening occurs.

Dr. Barrett—The idea so very frequently expressed in all the medical works that the eruption of the teeth, during the period of dentition, causes such irritation and derangement of the whole digestive system is, I think, an erroneous one. In health the eruption of the teeth is a perfectly physiological process. It should no more effect the general condition of the system than the growing of one's finger nails. It is entirely physiological. Why is it then that people are particularly liable to stomach diseases just at that time? Because it is between hay and grass with the child, to use a common expression. It is just at the time when the digestive organs are undergoing a change from that simple pabulum for which they were primarily adapted, to a grosser, heavier diet. During this change children are peculiarly liable to gastritis and derangements of that kind. This is not caused by the eruption of the teeth. It comes from mal-nutrition, indigestion, or other nutritive disturbances. The irritation caused by the eruption of the teeth has no more to do with it than the growing of the hair upon one's head. I believe that there is a hundred times more injury done by the cutting of the gum than by leaving it alone. If the child is in anything like a healthy state there is no trouble about its teething. When there is sufficient pressure upon the gum there will be absorption, and the tooth will come through easily. In cutting, nineteen times out of twenty, and I don't know but oftener, it is done at the wrong time, and a cicatricial tissue is formed which only increases the difficulty in the eruption of the teeth. If I ever did cut the gum at all, I should slip the point of a pen-knife under it and cut upwards and outwards; I have seen children that were extremely ill, nearly in convulsions. I remember one particular case where I felt very confident that the cutting of the gum would relieve the difficultty, because of the extreme apparent tension. I had read so much about the pressure producing convulsions. So I used the lancet, but it did not do any good. It was simply a case of indigestion or gastralgia. The cutting of the gum was not of the slightest benefit to the child.

Dr. Brophy—I disagree entirely with what Dr. Barrett has said in regard to lancing of the gum. Furthermore, I am surprised that he should state, as a physiologist, that the breaking through a cicatricial tissue is far more difficult than breaking through ordinary tissues. A cicatrice in all cases is the most easy and yielding of any. A cut which has healed is a great deal more yielding than the original tissue. Even if the gums do heal up and a cicatrice forms, cut them again and relieve the parts of the blood. I am convinced that the pressure upwards by the teeth against the tissues produces a disturbance of the nervous system, and that is what brings about the trouble. That this simple operation on the gums has been productive of a great deal of good and saved the lives of hundreds of children, I have not a particle of doubt.

Dr. Barrett—I cannot subscribe to the idea that lancing the gum removes all the difficulties connected with dentition. If you are going to cut for the sake of depletion I will agree with you; but as to performing this operation as a cure for stomatitis, gastritis, etc., I do not subscribe to that. The trouble has its origin back of the inflammation and irritation, in those diseases and disarrangements of the stomach which produce convulsions and death itself in some cases.

Dr. Taft—Cases of difficult dentition have been referred to. It is from disturbed functions that the trouble arises. If the growing tooth comes as it should, and is properly supported in its growth, if the way is prepared for it as it advances, there will be no difficulty in the way of dentition later. The trouble is that there is a defect in nutrition, a disturbance of some sort. Usually the great difficulty is produced by pressure upon the nerves in the parts pressed upon. Now, I do not presume that the actual pressure of the tooth is the normal means of preparing its way. I think the normal process should be independent of the pressure of the teeth. I do not think the tooth struggles through the surrounding tissue. I think the tissue is absorbed away in anticipation of the coming of the tooth. The gum affords but little resistance when everything is in a healthy state, for by a physiological function the way is prepared. If there is any disturbance of this process, then the tooth comes in contact with the tissue and pressure is sometimes the result as we know. The irritation consequent upon the failure of this work of preparation may result in one of two ways. The pressure of the teeth may produce irritation and inflammation. The pressure in the blood vessels may operate to produce a nervous disturbance through the parts. That is one way in which pain is produced. Another way, and a more serious one as it seems to me, is when the pressure becomes greater, and the blood is forced out of the vessels. Then the pressure upon the parts is materially augmented. In such cases there will be more frequent reflex nervous influence exhibited one way or the other, on the brain, the stomach, the digestive apparatus or some other.—*Transactions Michigan Dental Society.*

---

Charcot reports some very interesting cases of "Hysterical Contracture of Traumatic Origin" in *Le Progres Medical.* One of his patients at the Salpetriere was a delicate girl of sixteen, whose father had died of general progressive paralysis. One of her father's brothers was almost an idiot. This, Charcot thinks, shows very well the neuropathic "heredity of transformation," *i.e.*, the ancestors having suffered from nervous diseases other than, but allied to hysteria. This girl's left hand exhibited flexion of the first phalanges upon the metacarpus, the fingers being tightly squeezed together. This deformity had been permanent for one year. It was due to simple muscular rigidity. The left hand was colder than the right, and had a bluish tint that evidenced vaso-motor disturbance. Over a year ago she had an insignificant cut on her hand that healed in four or five days, and the patient having what may be termed a *contracture diathesis*, the above deformity resulted. When this diathesis exists even sudden movements, such as throwing a stone, are followed by contractures very marked and obstinately resisting treatment. Moreover, this girl had left hemianalgesia with left sensory hemi-anæsthesia ; and by excluding the existence of cerebral lesions, alcohol or lead poisoning, it was left us only to regard it as one of the many manifestations of purely classical hysteria.

